# Giant thymoma successfully resected via median sternotomy and anterolateral thoracotomy: a case report

**DOI:** 10.1186/s13019-018-0711-z

**Published:** 2018-04-10

**Authors:** Yoko Azuma, Hajime Otsuka, Takashi Makino, Satoshi Koezuka, Yoichi Anami, Sota Sadamoto, Megumi Wakayama, Naobumi Tochigi, Kazutoshi Shibuya, Akira Iyoda

**Affiliations:** 1 Division of Chest Surgery, Toho University School of Medicine, Tokyo, Japan; 2 Department of Surgical Pathology, Toho University School of Medicine, Tokyo, Japan

**Keywords:** Giant thymoma, Median sternotomy, Anterolateral thoracotomy, Abbreviations, CT computed tomography, WHO World Health Organization, FDG-PET F18-fluorodeoxyglucose positron emission tomography, AchR acetylcholine receptor, MG myasthenia gravis

## Abstract

**Background:**

Some patients with thymoma present with a very large mass in the thoracic cavity. Although the most effective treatment for thymoma is surgical resection, it is difficult to perform because of the size of the tumor and the infiltration of tumor into the surrounding organs and vessels. We report a patient with a giant thymoma that was completely resected via a median sternotomy and left anterolateral thoracotomy.

**Case presentation:**

A 63-year-old woman presented with a mass in the left thoracic cavity that was incidentally found on a chest X-ray. Chest computed tomography revealed a giant mass (16 × 10 cm) touching the chest wall and diaphragm and pressed against the heart and left upper pulmonary lobe. Complete resection was performed via a median sternotomy and left anterolateral thoracotomy. The tumor was histologically diagnosed as a WHO type B2 thymoma, Masaoka stage II.

**Conclusions:**

Giant thymomas tend to grow expansively without invasion into surrounding organs and vessels. Surgical resection that employs an adequate approach must be considered, regardless of the size of the tumor.

## Background

Thymic epithelial neoplasms are commonly located in the anterior mediastinum. The tumors typically show slow-growing behavior. Patients present with various clinical signs and symptoms that are associated with expansion of the tumor; the most effective treatment modality is surgery [1]. Giant thymomas are very rare and difficult to resect because of the size of the tumor and involvement of surrounding organs. Here, we report a case of giant thymoma that was completely resected via a median sternotomy and anterolateral thoracotomy.

## Case presentation

**Fig. 1:**
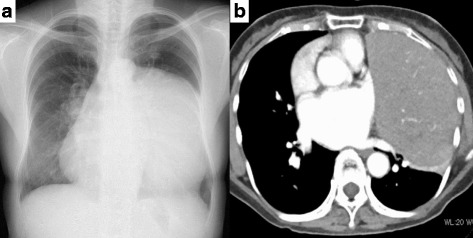
**a** Chest X-ray shows a very large mass in the left middle and lower lung fields. **b** Chest computed tomography shows a tumor (16 × 10 × 7 cm) in direct contact with a large area of the chest wall and diaphragm, and pressing on the heart and left upper pulmonary lobe

A 63-year-old woman was seen at a local hospital for chest bruising secondary to an accident. Chest radiography revealed an abnormal shadow in the left middle and lower lung fields (Fig. 1a). The finding was diagnosed as an anterior mediastinal mass, and the patient was referred to our hospital for treatment. The patient had a history of bronchial asthma. Chest computed tomography (CT) showed a giant, well defined mass measuring 16 × 10 cm in the left thoracic cavity. Contrast-enhanced CT revealed a mass with heterogenous enhancement that was in direct contact with a large area of the chest wall and diaphragm and pressed against the heart and left upper pulmonary lobe (Fig. 1b). F18-fluorodeoxyglucose positron emission tomography (FDG-PET) showed abnormal FDG uptake with a maximum standardized uptake value of 3.77. Laboratory examination showed high serum levels of acetylcholine receptor (AchR) antibody (5.3 nmol/L), although the patient had not complained of any symptoms suggestive of myasthenia gravis (MG). A thymoma was suspected, and surgical resection was recommended. Since the CT findings suggested adhesions between the tumor and the chest wall, diaphragm, pericardium, and left lower lobe of the lung, or infiltration by the tumor, we performed a median sternotomy and left anterolateral thoracotomy without changing position of the patient for the resection.

**Fig. 2:**
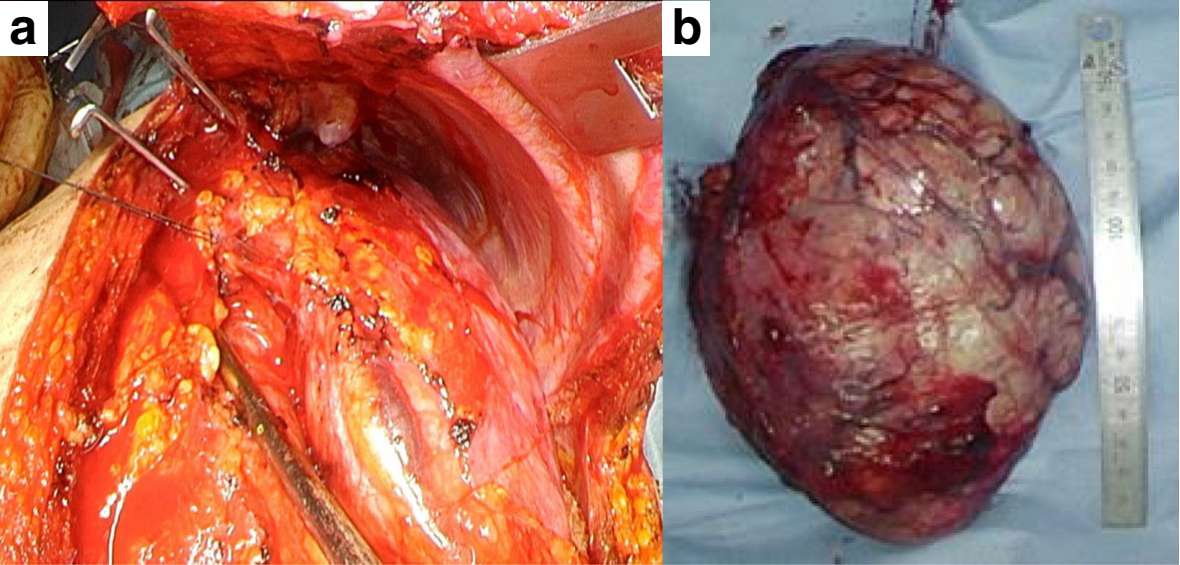
**a** Intraoperative view of the giant thymoma. The tumor was excised from the anterior mediastinal fat tissue and thymus, and occupied approximately half of the left pleural cavity. **b** The resected specimen was 16.5 × 11.8 × 7 cm in size

A 16-cm anterolateral incision was made in the fifth intercostal space. The tumor occupied approximately half of the left pleural cavity (Fig. 2a). It was excised from the anterior mediastinal fat tissue and thymus. Fibrous adhesions extending from the tumor to the left lung and diaphragm were sharply peeled off, and the tumor was resected without involvement of the pericardium or trunk of the pulmonary artery (Fig. 2b). The findings of an intraoperative frozen section of the tumor were diagnosed as thymoma. An extended total thymectomy was thus performed through the same incision used for the resection.

**Fig. 3:**
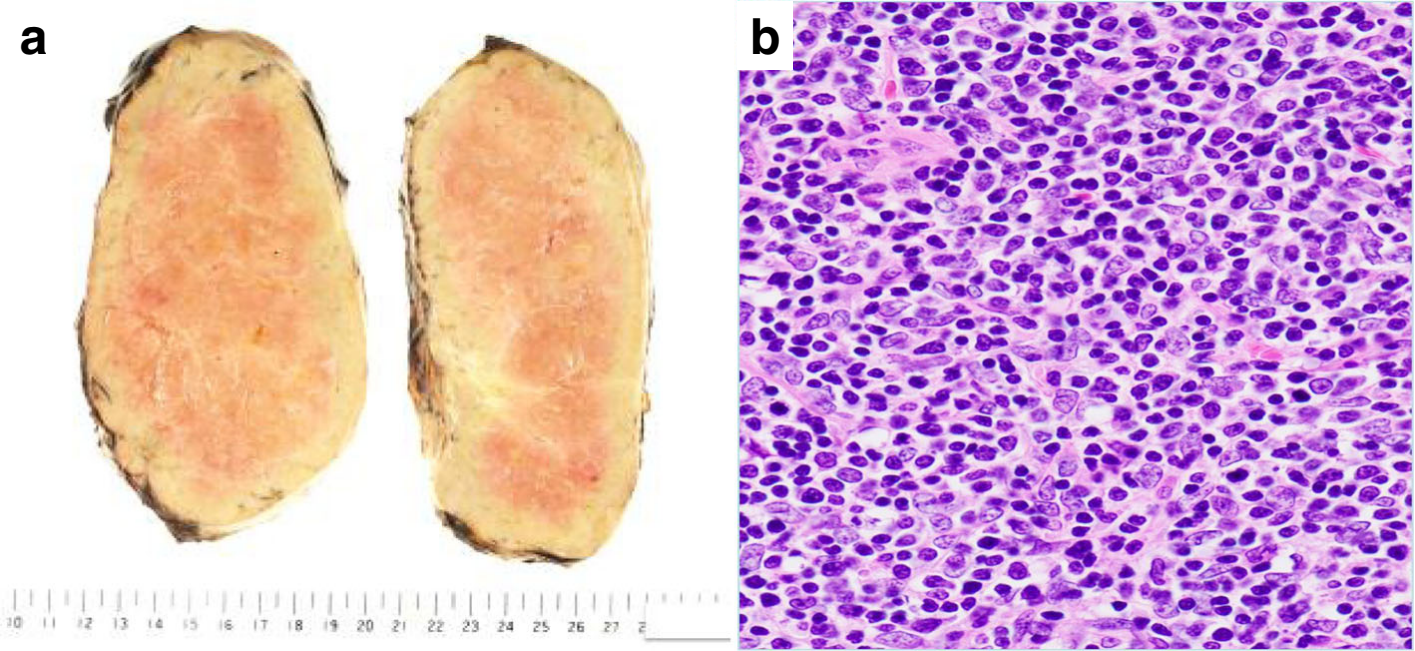
**a** Gross pathology of the tumor. The cut surface of tumor was colored red and yellowish, and appeared to be lobulated internally. **b** Histological findings of the tumor. The tumor was composed of a lymphocyte associated area and was diagnosed as a WHO type B2 thymoma with capsular invasion (Masaoka stage II)

The resected specimen was 16.5 × 11.8 × 7 cm, and showed a well encapsulated tumor with lobules separated by fibrous bands (Fig. 3a). Microscopic examination revealed the tumor to be composed of a lymphocyte associated area (Fig. 3b), and the findings were diagnosed as World Health Organization Type B2 thymoma with capsular invasion (Masaoka stage II). The patient’s postoperative course was uneventful, and she remains free of recurrence and signs and symptoms associated with MG 1 year after the surgery.

## Discussion

Thymomas account for approximately 20% to 25% of all mediastinal masses in individuals of all ages and 47% of all mediastinal masses in adults [2–4]. Giant thymomas in adults are very rare. Table 1 summarizes the characteristics of the patients with giant thymomas in previously published reports and the characteristics of our patient [3, 5–14]. Thymomas typically grow slowly and expansively, and most patients with thymoma, including our case, are asymptomatic [2–4]. But patients can manifest various signs and symptoms, including chest pain, dyspnea, and other upper respiratory problems due to tumor growth [15]. Table 1 shows that many cases of giant thymoma presented with signs and symptoms related to involvement of the thymoma with adjacent organs.

**Table 1:** Characteristics of patients with giant thymoma

Author	Year	Age/Sex	Symptoms	Tumor size (cm)	Histology (WHO)	Masaoka Stage	Surgical Approach	Resection Completeness
Takanami [3]	1999	62 M	asymptom	13 × 10 × 10	A	I	median + right anterolateral	R0
Santoprete [5]	2007	73 F	chest pain	12 × 14 × 12	AB	II	left clamshell	R0
Yamazaki [6]	2007	58 F	palpitation	20 × 14 × 8	A	III	right anterolateral	R0
Fazliogullari [7]	2012	28 M	dyspnea	17 × 12 × 7	AB	II	median	R1
Takenaka [8]	2012	61 M	cough	18 × 14 × 11	AB	II	median	R0
Filosso [9]	2012	74 F	dyspnea	14 × 13 × 8	AB	II	right posteroalateral	R0
Spartails [10]	2012	83 F	kyphosis	20 × 11 × 2	B1	I	median	R0
Aydin [11]	2012	23 F	chest pain	21 × 7 × 7	B1	II	right posteroalateral	R0
Saito [12]	2015	45 M	asymptom	13 × 10	AB	I	right anterolateral	R0
Zhao [13]	2016	46 M	dyspnea	19 × 16 × 15	AB	II	left hemic lamshell	R0
Alexiev [14]	2016	49 F	cough	10 <	AB	I	left anterolateral	R0
Present case	2017	63 F	asymptom	16 × 10 × 7	B2	II	median + left anterolateral	R0

Surgical resection is generally accepted to be the most effective treatment for thymoma, and complete resection is an important prognostic indicator of long-term outcome [16]. Large size is a poor prognostic factor in thymoma, and complete resection largely contributes to a successful treatment outcome for patients with giant thymoma. Although thymomas can present as huge masses, tumor stage may not always be correlated with tumor size [17]. Interestingly, most giant thymomas have been found to be low grade histologically, without invasion into the surrounding organs and vessels, and have been completely resected (Table 1). The noninvasiveness of giant thymomas might account for their presentation as very large tumors.

A median sternotomy is the standard procedure for resecting a thymoma of normal size, but the procedure is controversial for giant thymoma (Table 1). A clamshell incision was used for an emergency operation for a patient with shock due to bleeding of a thymoma [5]. This approach enables access to both hila and the pleural cavity. One patient underwent resection via a hemiclamshell approach, which allows access to the upper thoracic cavity [13]. Both the clamshell and hemiclamshell incisions are more invasive than other approaches. A posterolateral approach was used for 2 patients [9, 11]. This approach is suitable for a tumor that extends to the inferior cavity, but a second surgery is needed to confirm complete thymectomy. A median sternotomy is suitable for patients with possible invasion of the innominate vein [7, 8], but access to the hila or posterior thorax can be difficult for cases of giant thymomas. Three patients with giant thymoma underwent resection via the anterolateral approach, which allows extension of the incision to include a posterolateral or hemiclamshell approach [6, 12, 14]. Only one patient with giant thymoma underwent resection via a median sternotomy and anterolateral thoracotomy [3], which was the approach we used for our patient. This approach allows wide access to the tumor and involved organs, regardless of their location in the thoracic cavity.

Some thymoma patients develop MG after thymectomy (“post-thymectomy MG”) regardless of whether or not they have a history or signs or symptoms of MG. Post-thymectomy MG develops in 1.0% to 28% of thymoma patients who have undergone thymectomy [18–23]. Previous reports showed that elevated preoperative serum AchR antibody levels and World Health Organization type B thymoma were risk factors for post-thymectomy MG [24, 25]. Our case corresponds to patients at high risk post-thymectomy MG, and requires careful follow-up for early detection of MG.

## Conclusions

We reported a rare case of giant thymoma that was successfully resected via median sternotomy and left anterolateral thoracotomy. Giant thymomas tend to be low-grade tumors that do not infiltrate adjacent organs and vessels. For successful treatment of giant thymoma, curative surgical resection must be considered, regardless of tumor size.
